# Surface antigens and potential virulence factors from parasites detected by comparative genomics of perfect amino acid repeats

**DOI:** 10.1186/1477-5956-5-20

**Published:** 2007-12-20

**Authors:** Niklaus Fankhauser, Tien-Minh Nguyen-Ha, Joël Adler, Pascal Mäser

**Affiliations:** 1 University of Bern, Institute of Cell Biology, Baltzerstrasse 4, CH-3012 Bern, Switzerland; 2 Pädagogische Hochschule Bern, Gertrud Woker Strasse 5, CH-3012 Bern, Switzerland

## Abstract

**Background:**

Many parasitic organisms, eukaryotes as well as bacteria, possess surface antigens with amino acid repeats. Making up the interface between host and pathogen such repetitive proteins may be virulence factors involved in immune evasion or cytoadherence. They find immunological applications in serodiagnostics and vaccine development. Here we use proteins which contain perfect repeats as a basis for comparative genomics between parasitic and free-living organisms.

**Results:**

We have developed Reptile , a program for proteome-wide probabilistic description of perfect repeats in proteins. Parasite proteomes exhibited a large variance regarding the proportion of repeat-containing proteins. Interestingly, there was a good correlation between the percentage of highly repetitive proteins and mean protein length in parasite proteomes, but not at all in the proteomes of free-living eukaryotes. Reptile combined with programs for the prediction of transmembrane domains and GPI-anchoring resulted in an effective tool for in silico identification of potential surface antigens and virulence factors from parasites.

**Conclusion:**

Systemic surveys for perfect amino acid repeats allowed basic comparisons between free-living and parasitic organisms that were directly applicable to predict proteins of serological and parasitological importance. An on-line tool is available at .

## Background

Repetitive amino acid subsequences in polypeptides are of interest regarding the function as well as the evolution of proteins. At least 14% of all proteins contain internal repeats, the proportion being somewhat lower in prokaryote and higher in eukaryote proteomes [1]. Multicellular eukaryotes in particular, possess numerous adhesion proteins of repetitive nature in the extracellular matrix. Other highly repetitive proteins are those of the cytoskeleton [1,2]. Typical motifs involved in protein-protein interaction are the tetratricopeptide repeat (34 aa), armadillo (47 aa), ankyrin (33 aa), and the leucine-rich repeat (about 20 aa) [3]. Several tools are available for the detection of repeats in proteins: Radar [4,5], Repro [6,7], Internal Repeats Finder [8,9], TRIPS [10,11], Trust [12,13], Davros [14], RepSeq [15,16], REP [2,17], Repper [18,19], and ProtRepeatsDB [20,21]. Apart from simply counting repetitive occurrences of amino acid subsequences in polypeptides, repeats can be detected by self-alignment or – if they are evenly distributed – by Fourier transform. Here we present Reptile, a simple tool for quantitative proteome-wide surveys of perfect amino acid repeats, and its use for the prediction of surface antigens and virulence factors from parasites.

Pathogenic bacteria as well as eukaryotic parasites often possess surface proteins of repetitive nature, presumably to protect themselves against their hosts' defence responses [22,23]. Examples are the procyclins of the sleeping sickness parasite *Trypanosoma brucei *with over twenty Glu-Pro (EP-type), respectively five Gly-Pro-Glu-Glu-Thr (GPEET-type) repeats [24,25], the circumsporozoite protein of the malaria parasite *Plasmodium falciparum *with around forty Asn-Ala-Asn-Pro (NANP) repeats [26], or SdrE from *Staphylococcus aureus*, a determinant of staphylococcal sepsis with 83 Ser-Glu (SE) repeats [27]. Such short, perfect repeats are usually very immunogenic. They may serve for serological diagnostics – the presence of repeat-directed antibodies in the serum indicating infection – as is the case with PfHRP2 [28], a malaria antigen with over fifty Ala-His-His (AHH) repeats. Repetitive amino acid sequences also find applications in synthetic vaccines [29]. Furthermore, repeat-containing proteins from parasites may be virulence factors involved in immune evasion, cytoadherence, stress resistance, or biofilm formation [30-35]. The completion of the genome sequencing projects for *P. falciparum*, *T. brucei*, *Leishmania major*, and other parasites now permits systemic approaches to repeat-containing proteins. Here we identify all proteins from pathogens that contain repeats and use them for comparative genomics between parasitic and non-parasitic species. All data and programs are freely accessible via the world-wide web.

## Results and Discussion

### Probabilistic description of perfect repeats with Reptile

In order to scan whole proteomes for repeat-containing proteins, we created the tool Reptile. It uses a "brute-force" algorithm that detects all perfect repeats and enables direct calculation of a P-value. For each input sequence, Reptile generates all possible substrings from length 2 to a user-defined maximum (the default is 20) and counts their occurrences. After removing redundant repeats that are contained within longer ones, the repeated sequences are returned by ascending P-value. The probability P to find at least n repeats of length r in a random sequence of length L (with nr ≤ L ≤ n20^r^) equals the number of possible sequences that contain the desired repeat, divided by the total number of possible sequences (20^L^).

$${\text{P}}^{*}(\text{n,r,L})=\frac{20^{\text{r}}20^{\text{L-nr}}}{20^{\text{L}}}\left(\begin{array}{c} \text{L-nr+n}\\ \text{n} \end{array}\right)=20^{\text{-r}(\text{n-}1)}\left(\begin{array}{c} \text{L-n}(\text{r-}1)\\ \text{n} \end{array}\right)$$

Where 20^r ^is the number of possible repeat sequences, 20^L-nr ^the number of possible sequences around the repeats, and the binomial equals the number of ways to place the n repeats in L. P* is an overestimate because the sequences with more than n repeats are counted too often. Taking this into account gives the correct formula for P:



Where *i *counts from n to the maximal number of repeats (L/r), switching signs with every increment according to the inclusion-exclusion principle [36]. For practical purposes calculation of P*, the first summand of P, is sufficient since further summands decrease rapidly with increasing number of repeats. Reptile returns all repeats below a user-defined cut-off P-value (the default is 10^-5^, corresponding to an expectancy of one false positive in 100'000 sequences). Direct repeats are marked. The P-value being independent of the actual sequence of a repeat, Reptile also returns a measure of whether a detected repeat consists of rare or frequent amino acids. This "Amino acid abundance measure" (AM) was defined as follows:

$$\text{AM}(\text{repeat})=\log_{10}(20^{\text{r}}\prod_{\text{i=1}}^{\text{r}}f_{\text{i}})$$

Where r is the length of the repeat and *f*_i _is the frequency in the corresponding proteome – respectively set of sequences submitted by the user – of the amino acid at position i of the repeat. AM is symmetric to zero, negative values indicating that a repeat predominantly consists of rare amino acids (and vice versa). Reptile is running on-line [37] and accepts batch input of up to 50,000 sequences in any of the commonly used formats.

Compared to other repeat-prediction programs (Table 1) the main strengths of Reptile are its quantitative assessment of the detected repeats and its infallibility regarding short perfect repeats, such as they occur in antigens from parasites. Reptile will spot in a given protein all recurring subsequences from length two to twenty, even if they are dispersed. In contrast to programs implementing self-alignment, however, Reptile does not properly recognize degenerate repeats. Though proteins harbouring degenerate repeats also exhibit low P-values and will not go unnoticed, Reptile will not identify the basic repetitive unit but several shorter ones contained within. Other programs (Table 1) should be used when studying large repeat regions or imperfect, diverging repeats.

**Table 1:** Comparison of programs for the detection of repetitive subsequences in proteins

**Program**	**Method used**	**Detection of degenerate repeats**	**Calculation of a P-Value**	**Analysis of whole Proteomes**	**%Hits found in SwissProt**	**Detection of T. brucei procyclin**^1^
Reptile	Hashing^2^	No	Yes	Yes	15^3^	Yes
REP [2]	Profiles of known repeats	Yes	No	No	1.1	No
RADAR [5]	Alignment	Yes	No	No	28	Yes
REPRO [7]	Alignment	Yes	No	No	n.a.	Yes
Internal Repeats finder [8]	Alignment	Yes	Yes	No	14	No
TRIPS [9]	Fourier transform	Yes	No	No	12	No
RepSeq [10]	Hashing	Yes	Yes	Yes	n.a.	Yes
ProtRepeatsDB [11]	Mixed	Yes	Yes	Yes	n.a.	Yes
Repper [12]	Fourier transform	Yes	No	No	n.a.	No

^1^The *T. brucei *surface protein (GenBank accession AAK62893) with five GPEET repeats [25] was used for benchmarking.

^2^Word count using a hash table.

^3^Using P < 0.001 (same as for Internal Repeats Finder).

### Genome-wide surveys for highly repetitive proteins

**Figure 1:**
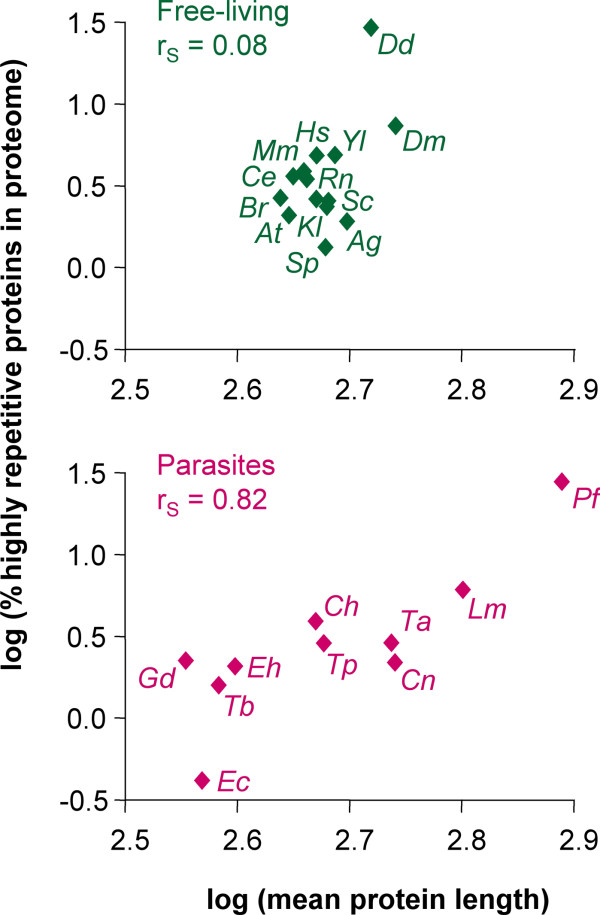
**Comparative genomics of repeat-containing proteins**. Double logarithmic plot of the percentage of highly repetitive (P < 10^-10^) proteins vs. mean protein length of eukaryotic proteomes. Ag, *A. gambiae*; At, *A. thaliana*; Br, *B. rerio*; Ce, *C. elegans*; Dd, *D. discoideum*; Dm, *D. melanogaster*; Hs, *H. sapiens*; Kl, *K. lactis*; Mm, *M. musculus*; Rn, *R. norvegicus*; Sc, *S. cerevisiae*; Sp, *S. pombe*; Yl, *Y. lipolytica*; Ch, *C. hominis*; Cn, *C. neoformans*; Ec, *E. cuniculi*; Eh, *E. histolytica*; Gd, *G. duodenalis*; Lm, *L. major*; Pf, *P. falciparum*; Ta, *T. annulata*; Tb, *T. brucei*; Tp, *T. parva*; r_S_, Spearman coefficient.

We defined highly repetitive proteins as proteins that contain perfect repeats of a P-value below 10^-10^. Reptile was used to screen for such proteins in predicted proteomes from fully sequenced genomes. The median proportion of highly repetitive proteins was 2.7% in eukaryote proteomes and 0.43% in prokaryotes, confirming the notion [1] that eukaryotes possess more repetitive proteins than bacteria (p < 0.0001, Mann-Whitney test). The more repeats a protein has, the longer it becomes. In eukaryotic proteomes the percentage of highly-repetitive proteins correlated to some degree with the mean protein length (Spearman coefficient r_S _= 0.51, p = 0.011). When distinguishing free-living from (endo)parasitic eukaryotes (Table 2), it was evident that the correlation was caused entirely by the latter. Obligate parasites exhibited a good correlation between highly-repetitive proteins and mean protein length (r_S _= 0.82, p = 0.003) while free-living eukaryotes showed no correlation at all (Figure 1). The finding that the percentage of highly repetitive proteins predicts average protein length only in parasite proteomes reflects the significance of repeat-containing proteins for survival in the host, possibly counterbalanced by a selective pressure on parasites for shorter proteins [38].

**Table 2:** Eukaryotic proteomes analyzed

**Organism**	**Kingdom**	**Type**	**Proteins**
*Homo sapiens*	Metazoa	F	38220
*Mus musculus*	Metazoa	F	35593
*Arabidopsis thaliana*	Viridiplantae	F	34554
*Caenorhabditis elegans*	Metazoa	F	22431
*Drosophila melanogaster*	Metazoa	F	16239
*Brachydanio rerio*	Metazoa	F	15647
*Anopheles gambiae*	Metazoa	F	13486
*Dictyostelium discoideum*	Protozoa	F	13017
*Rattus norvegicus*	Metazoa	F	11987
*Yarrowia lipolytica*	Fungi	F	6525
*Saccharomyces cerevisiae*	Fungi	F	5810
*Kluyveromyces lactis*	Fungi	F	5326
*Schizosaccharomyces pombe*	Fungi	F	5009
*Entamoeba histolytica*	Protozoa	P	9772
*Giardia duodenalis*	Protozoa	P	9646
*Trypanosoma brucei*	Protozoa	P	9210
*Leishmania major*	Protozoa	P	8010
*Cryptococcus neoformans*	Fungi	P	6569
*Plasmodium falciparum*	Protozoa	P	5283
*Theileria parva*	Protozoa	P	4071
*Cryptosporidium hominis*	Protozoa	P	3886
*Theileria annulata*	Protozoa	P	3790
*Encephalitozoon cuniculi*	Fungi	P	1909

F, free-living; P, endoparasitic.

The eukaryote with the largest proportion of highly repetitive proteins, *Plasmodium falciparum *with 28%, and that with the smallest one, *Encephalitozoon cuniculi *with 0.42%, were both obligate parasites. The same applied to prokaryotes, where the highest proportions of highly repetitive proteins were exhibited by *Mycobacterium bovis *(3.0%), *M. tuberculosis *(2.9%) and *Parachlamydia *sp. (2.7%), and the lowest ones by *Bacillus anthracis *(Porton strain, 0.02%) and *Streptococcus pyogenes *(SSI strain, 0.05%) – however, it must be noted that with bacteria, the available genome sequences are biased towards pathogenic species. The most repetitive protein from eukaryotes was a hypothetical protein from the sleeping sickness parasite *T. brucei*, followed by the 11-1 gene product from *P. falciparum*, a known malaria antigen of more than 1 MD size [39]. The most repetitive prokaryotic protein was a predicted cell wall surface anchor family member from *Streptococcus pneumoniae*, the leading cause of pneumonia. Table 3 summarizes these and other highly repetitive proteins identified from pathogens, emphasizing on sequences with experimentally verified expression. The genome-wide surveys yielded other known virulence factors such as proteophosphoglycans of *Leishmania *[40] or PGRS (polymorphic GC-rich repetitive sequence) proteins of *Mycobacterium*, an antituberculosis vaccine candidate [41]. The presence of avirulence proteins from phytopathogenic bacteria among the most repetitive proteins indicates that repeats also serve to specifically trigger host defence responses. Remarkably repetitive are also the ice nucleation proteins of plant pathogens. Table 3 also shows examples of previously undescribed proteins. The complete datasets on repeat-containing proteins from 49 eukaryotes and 193 prokaryotes are accessible on-line in the archive REPository [37].

**Table 3:** A selection of the most repetitive proteins from pathogens

**Name, accession**	**Sp**	**L**	**Repeat**	**pP**
Hypothetical protein, Tb927.1.1740	Tb	7154	132 × LAEESQQHTARSEADIDE	2806
Gene 11-1 protein*, Q8I6U6	Pf	10589	967 × EEV	2457
Conserved protein, LmjF29.0110	Lm	3418	146 × AEEQARR	1080
Proteophosphoglycan-like, LmjF35.0550	Lm	2425	105 × SSSSSAPSA	1052
Putative antigen*, Tb04.29M18.750	Tb	4455	66 × NEQYETLQRTNAA	958
Gb4*, Tb09.160.1200	Tb	8214	35 × VVIIDCRLGSLLIDYKVI	701
Hypothetical protein, Chro.50162	Ch	1589	84 × KKDAP	407
Hypothetical protein, Q8I455	Pf	2349	67 × LKEEER	389
Interspersed repeat antigen*, Q8I486	Pf	1720	67 × QEPVT	313
Putative antigen 332*, Q8IHN3	Pf	5507	144 × EEI	274
Cell wall surface anchor family, Q97P71	Spn	4776	1074 × SAS	3418
Cell surface SD repeat protein, Q88XB6	Lpl	3360	796 × DS	1619
Hypothetical protein, Q8E473	Sag	1310	106 × TSAS	447
Putative peptidoglycan-bound, Q8Y697	Lmo	903	78 × ADADA	403
Avirulence protein, Q5GYF3	Xor	1790	20 × ETVQRLLPVLCQDHGLTP	401
Serine/threonine-rich antigen, Q99QY4	Sau	2271	163 × STS	391
PE-PGRS family, PG54_MYCTU	Mt	1901	136 × GAG	326
Structural toxin RtxA, Q5X7A6	Lpn	7679	29 × RFEDDGPVV	247
Ice nucleation protein, Q8PD38	Xca	1333	52 × GYGST	242
PPE family protein, Q6MX44	Mtu	3300	95 × NTG	184

Eukaryotic proteins (top) whose expression is confirmed by the presence of expressed sequence tags (EST) in GenBank are marked with an asterisk. L, length; pP, negative logarithm of the P-value; Sp, species (Ch, *C. hominis*; Lm, *L. major*; Pf, *P. falciparum*; Tb, *T. brucei*; Lmo, *Listeria monocytogenes*; Lpl, *Lactobacillus plantarum*; Lpn, *Legionella pneumophila*; Mtu, *M. tuberculosis*; Sau, *S. aureus*; Spn, *S. pneumoniae*; Sag, *Streptococcus agalactiae*; Xca, *Xanthomonas campestris*; Xor, *X. oryzae*).

### Amino acid composition of the repeats

**Figure 2:**
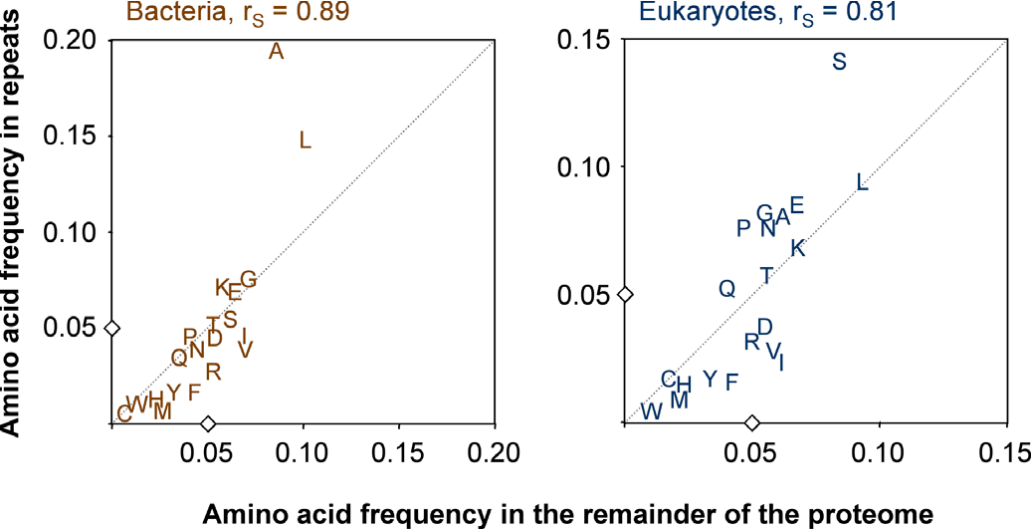
**Amino acid composition of the repeats**. For each amino acid, the frequency in the repeats of P < 10^-10 ^is plotted vs. its frequency in the remainder of the proteome (r_S_, Spearman coefficient). Data are pooled for bacteria (n = 193) and eukaryotes (n = 49). The small diamonds at 0.05 mark the expected frequency for random distribution, the diagonal represents equal frequency in the repeats as in the remainder of the respective proteome. Complete datatables including standard deviation are provided as a supplementary file [Additional file 1].

**Figure 3:**
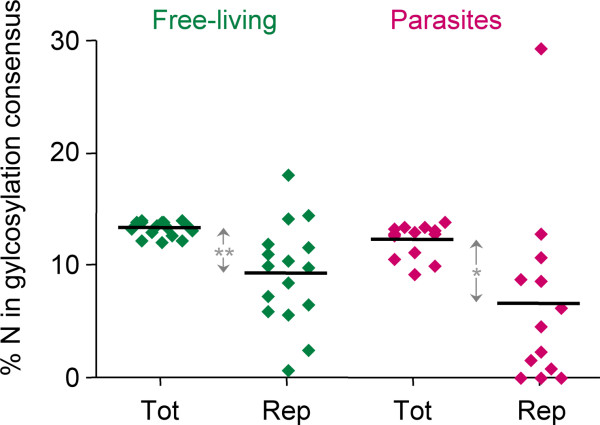
**Potential N-glycosylation sites in the repeats**. The percentage of asparagines that are in glycosylation consensus (Asn-not Pro-Ser/Thr) is plotted for repeats of P < 10^-10 ^and for the remainders of the respective proteomes. Bars indicate the median. The organism with 30% of asparagines in the repeats in N-glycosylation consensus is *T. brucei*.

To further characterize the repeats, we investigated which amino acids are over- or underrepresented in repeats of P < 10^-10 ^compared to the rest of the respective proteome. Overall, the amino acid composition of the repeats was more biased in eukaryotes than in bacteria (Figure 2). Small amino acids occurred more frequently in the repeats than large ones in both eukaryotes and prokaryotes. Hydrophobic residues were underrepresented in the repeats, with the exception of leucine, which in bacterial repeats was even overrepresented (p < 0.0001, two-tailed Wilcoxon signed rank test). Strongly overrepresented in the repeats were alanine (p < 0.0001) in bacteria and serine (p = 0.0001) in eukaryotes (Figure 2). Thus "cheap" amino acids seem to be preferred over energetically expensive ones. Interestingly, asparagine was overrepresented in the repeats from eukaryotes (p = 0.057) but not from bacteria (Figure 2), suggesting that asparagines might be preferentially glycosylated in repeats. Contrary to expectation though, the probability of an asparagine to be in N-glycosylation consensus was significantly lower in repeats than in non-repetitive sequences (Figure 3). This was the case for free-living eukaryotes (p = 0.004) as well as for parasites (p = 0.027). The only exception was *T. brucei*, where the likelihood of an asparagine to be in N-glycosylation consensus was three-fold higher in repetitive than in non-repetitive sequences (Figure 3).

### Prediction of repetitive surface antigens

**Figure 4:**
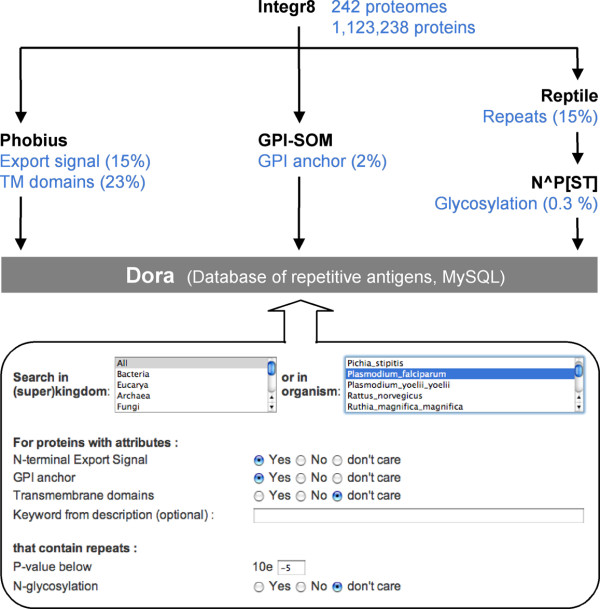
**Flowchart to Dora, database of repetitive antigens**. Reptile, Phobius [20], and GPI-SOM [43] are integrated into an automated pipeline for the classification of proteins (top). The data are stored in a database that is accessible on-line [44] via the depicted interface (bottom). This allows user-defined Boolean queries for repeat-containing surface proteins.

In order to predict which of the repeat-containing proteins are at the cell surface, Reptile was combined with Phobius [42], a program for prediction of transmembrane domains and N-terminal export signals, and GPI-SOM [43], a program that predicts C-terminal GPI (glycosylphosphatidyl-inositol) anchor attachment sites. The three programs were run over all available proteomes predicted from completely sequenced genomes. The identified repeats were scanned for potential N-glycosylation sites. The combined output was stored in a relational database called Dora, the database of repetitive antigens, as outlined in Figure 4. At present, Dora contains data on 1,123,238 proteins from 242 different proteomes (among which 49 eukaryotic). A www interface [44] allows user-defined Boolean searches (Figure 4). With Dora, genome-wide prediction of potential surface antigens and virulence factors is straightforward. A search for repetitive membrane proteins in *P. falciparum *or *T. brucei *(Table 4) indeed returned important surface antigens and virulence factors: circumsporozoite protein (CSP), merozoite surface proteins (MSP), erythrocyte membrane proteins (EMP), glycophorin-binding proteins (GBP), apical membrane/erythrocyte binding antigen (MAEBL), ring-infected erythrocyte surface antigen (RESA), mature parasite-infected erythrocyte surface antigen (MESA) for malaria and for *T. brucei *the procyclins, cysteine-rich acidic membrane protein (CRAM), invariant surface glycoproteins (ISG) and even the variable surface glycoproteins (VSG), which contain a significant number of dipeptide repeats (mostly AA; to our knowledge the repetitive nature of VSG was not previously recognized). In addition to these known proteins there was a large number of uncharacterized ones, particularly from *P. falciparum *which possesses hundreds of extremely repetitive transmembrane proteins (not shown; please refer to Dora).

**Table 4:** Repetitive membrane proteins of *P. falciparum *(top) and *T. brucei *(bottom)

**Name, accession**	**Topology**	**Repeat**	**pP**
Hypothetical protein, Q8IJ50	GPI	16 × EESHNFYNPTH	184
Circumsporozoite protein, Q7K740	GPI	38 × ANPN	145
Merozoite surface protein 8, Q8I476	GPI	32 × NN	29
Liver stage antigen, Q8IJ44	1 TM	45 × AKEKLQEQQSDLEQER	839
Erythrocyte membrane protein 3, O96124	1 TM	61 × QQNTGLKNTP	665
Trophozoite antigen, Q8IFL9	1 TM	60 × NHKSD	287
Glycophorin-binding protein, Q8I6U8	1 TM	10 × DPEGQIMREYAADPEYRKHL	213
MAEBL, Q8IHP3	1 TM	19 × EEKKKADELKK	213
PF70 exoantigen, Q8IK15	3 TM	8 × TKKPSKYTMNLDSPLLKGSS	165
MESA, Q8I492	1 TM	94 × KE	97
PfEMP1, Q8I519	1 TM	16 × GGGGGS	77
RESA, Q8IHN1	1 TM	33 × EEN	63
Hypothetical protein, Tb11.02.2360	GPI	11 × TAVTDVNDNNSANTSNEDE	229
Hypothetical protein, Tb11.1550	GPI	12 × IIAHYC	68
Procyclin (EP-type), Tb10.6k15.0020	GPI	29 × PE	46
Hypothetical protein, Tb927.7.360	GPI	3 × DKEKTERTEVEEVPKKDPEG	45
Procyclin (GPEET-type), Tb927.6.510	GPI	6 × EETGP	24
VSG, Tb10.v4.0209	GPI	19 × AA	13
CRAM, Tb10.6k15.3510	1 TM	80 × ITGDCNETDDC	1050
Hypothetical protein, Tb927.3.5530	2 TM	49 × RLRAEEE	337
Hypothetical protein, Tb10.61.0660	3 TM	12 × NEEVPAGVSARRGGVAMSF	241
Procyclic surface glycoprotein, Tb10.26.0790	2 TM	5 × YGQPPPPQ	31
Invariant surface glycoprotein, Tb927.5.350	1 TM	18 × EA	12

TM, transmembrane domain; GPI, glycosylphosphatidyl-inositol anchor; pP, negative logarithm of the P-value. See text for full protein names.

New specific and robust tests are urgently needed for the diagnosis of sleeping sickness, malaria, tuberculosis, and other neglected diseases [45,46]. PCR not being applicable in the field, serology (i.e. the detection of parasite-specific antibodies) remains the principal method of detection for many tropical diseases. Dora provides a convenient portal for identification of candidate antigens for serological tests. In addition, it can be helpful for the selection of vaccine candidates. Dora returns the hits in Fasta format, which is suitable for subsequent bioinformatic analyses.

## Conclusion

Reptile's simple algorithm allows large-scale and quantitative description of perfect amino acid repeats. Originally designed to scan parasite proteomes for potential antigens and virulence factors, Reptile detects any protein of repetitive nature and thereby complements existing tools which work by self-alignment. Parasite proteomes vary considerably regarding the proportion of repetitive proteins, in contrast to those of free-living eukaryotes which all contain around 3% highly repetitive (P < 10^-10^) proteins. Furthermore, the proportion of highly repetitive proteins correlates with mean protein length in parasites but not in the proteomes of free-living eukaryotes, illustrating the importance of amino acid repeats for parasites.

Scanning the predicted proteomes of parasites for amino acid repeats returned a large number of interesting proteins. Particularly useful was the combination of Reptile with prediction of glycosylation sites, export signals, transmembrane domains and GPI-anchor attachment sites, carried out on more than one million proteins from 242 different organisms. All data are accessible on-line via Dora, database of repetitive antigens. The approach was validated against *T. brucei *and *P. falciparum*, where a Dora search returned the known surface antigens, virulence factors, and vaccine candidates plus many new, so far uncharacterized proteins.

## Methods

### Proteome files

Predicted proteome files were obtained from the Integr8 database of the European Bioinformatics Institute [47]. The download was automated with a Python script that periodically checks for newly available proteomes, respectively for updates to previous proteome files.

### Statistics

Statistical tests were performed with Prism 4.0 (GraphPad Software). Since the percentages of repeats in proteomes as well as the frequencies of amino acids were not normally distributed, non-parametric tests were used: Mann-Whitney test [48], Wilcoxon signed rank test [49], and Spearman correlation [50].

### Reptile

The repeat detection algorithm is described under Results. The program is written in C++ and the web-interface in Perl-CGI. Reptile uses sreformat from the HMMer package [51] to convert different input formats (Fasta, GenBank, EMBL, Swiss-Prot, PIR, GCG) to Fasta. Reptile runs on a vmware (virtual infrastructure) server. Availability and requirements:

Project name: Reptile

Project home page: 

Operating systems: Linux, Unix

Programming language: C++

Licence: GNU GPL

### Dora

A Python script periodically runs Reptile, GPI-SOM, and Phobius over all new or updated proteome files of Integr8. The results are stored in a MySQL database. For sake of simplicity, for each protein only the repeat with the lowest P-value is stored. A Perl script is used to interconvert Fasta format and SQL. The web interface of Dora is written in PhP. The database and all the programs run on the vmware server of the Informatics Services of the University of Bern.

## Competing interests

The author(s) declare that they have no competing interests.

## Authors' contributions

NF developed all software and generated all the data. TN designed the MySQL database and created the user interface of Dora. JA derived the formula for the calculation of the P-value. NF and PM conceived the study, wrote the manuscript, and designed the figures. All authors read and approved the final manuscript.

## Supplementary Material

Additional file 1 Amino acid frequencies. Additional file 1 is a MS Excel file with two tables on separate Worksheets. Table 1 contains the amino acid frequencies in the predicted proteomes of 29 eukaryotes and 198 bacteria; Table 2 contains the amino acid frequencies in the repeats of P < 10^-10 ^of the same proteomes. Click here for file
